# Validation of Pretreatment Methods for the In-Process Quantification of Foot-and-Mouth Disease Vaccine Antigens

**DOI:** 10.3390/vaccines9111361

**Published:** 2021-11-19

**Authors:** Ah-Young Kim, Sun Young Park, Sang Hyun Park, Jong Sook Jin, Eun-Sol Kim, Jae Young Kim, Jong-Hyeon Park, Young-Joon Ko

**Affiliations:** Center for FMD Vaccine Research, Animal and Plant Quarantine Agency, Gimcheon 39660, Korea; mochsha@korea.kr (A.-Y.K.); sun3730@korea.kr (S.Y.P.); shpark0205@korea.kr (S.H.P.); in75724@korea.kr (J.S.J.); kesol13@korea.kr (E.-S.K.); ivorikim@korea.kr (J.Y.K.); parkjhvet@korea.kr (J.-H.P.)

**Keywords:** foot-and-mouth disease (FMD), pretreatment, quantification, vaccine antigen, SE-HPLC

## Abstract

Foot-and-mouth disease (FMD), caused by the FMD virus (FMDV), is controlled by vaccine policy in many countries. For vaccine potency, the content of intact virus particles (146S antigens) is critical, and the sucrose density gradient (SDG) fractionation is the gold standard for the quantification of 146S antigens. However, this method has several drawbacks. Although size-exclusion high-performance liquid chromatography (SE-HPLC) was introduced to replace the classic method, its application is generally confined to purified samples owing to the interfering signals. Therefore, we aimed to develop optimal pretreatment methods for SE-HPLC quantification in less purified samples. Crude virus infection supernatant (CVIS) and semi-purified samples with PEG precipitation (PEG-P) were used. Chloroform pretreatment was essential to remove a high level of non-specific signals in CVIS, whereas it caused loss of 146S antigens without the distinctive removal of non-specific signals in PEG-P. Benzonase pretreatment was required to improve the resolution of the target peak in the chromatogram for both CVIS and PEG-P. Through spiking tests with pure 146S antigens, it was verified that the combined pretreatment with chloroform and benzonase was optimal for the CVIS, while the sole pretreatment of benzonase was beneficial for PEG-P.

## 1. Introduction

Foot-and-mouth disease (FMD), caused by the FMD virus (FMDV), is a highly contagious animal disease threatening the livestock industry [1]. The virus affects cloven-hoofed animals, resulting in characteristic vesicle formation in the mucosa around the foot and mouth [2]. To control this disease, vaccination policies utilizing inactivated FMD vaccines have been implemented worldwide. The intact virion of FMDV, comprising 60 copies each of four structural proteins and a single-stranded RNA genome [3], is called a 146S particle based on its sedimentation coefficient [4]. As 146S particles are so unstable that they can be easily dissociated into less immunogenic 12S particles under mild changes in pH or temperature [5], it is important to know the amount of intact particles remaining in the vaccine to guarantee vaccine efficacy [6]. Identifying the 146S content is also critical for in-process quality control during the FMD vaccine production process.

Originally, the sucrose density gradient (SDG) method was the gold standard for 146S particle quantification [7]. However, this classic method not only involves the preparation of sucrose gradient tubes, 4 h of ultracentrifugation and laborious manual operation but also has a limitation on the number of concurrently treatable samples. Therefore, new methods for FMD vaccine antigen quantification, such as a double antibody sandwich (DAS) enzyme-linked immunosorbent assay (ELISA) [7,8] and size-exclusion chromatography (SEC) [9,10,11], have been introduced to replace the classic SDG method. Although DAS-ELISA seems to be easy to use and quite reliable, to the best of our knowledge, there is no commercially available antibody that can selectively detect intact virions without nonspecific binding to the subunit proteins. Instead, size-exclusion high-performance liquid chromatography (SE-HPLC) analyses using gel columns have recently been recommended because of their high specificity, repeatability, and accuracy, in addition to the good correlation with classic SDG quantification results and user convenience [10,11,12].

Using this new methodology, manufacturers can check the 146S particle content during the FMD vaccine antigen production process in real time for quality control. However, it is difficult to quantify 146S particles without proper pretreatment as samples have different purities depending on the phase of the production process. In particular, optimized pretreatment techniques are critical for the SE-HPLC analyses of samples from upstream production processes that have many impurities [13].

Several previous studies have shown that the enzymatic digestion of nucleic acids is essential for SE-HPLC quantitative analyses of the FMD vaccine antigen-containing downstream samples, for example, aqueous extracts from complete vaccine [10,11,14]; however, it is still unknown whether the sole use of nuclease would be adequate to eliminate interfering substances for the SE-HPLC analyses of FMDV 146S particles in upstream samples.

Therefore, we aimed to find a rational pretreatment method for the SE-HPLC analyses of FMD vaccine antigens, depending on samples from different phases of the production process, using both upstream and downstream samples that are represented by a crude virus infection supernatant (CVIS) for the former and a polyethylene glycol (PEG) precipitate (PEG-P) for the latter.

## 2. Materials and Methods

### 2.1. Preparation of FMDV Samples

FMDV O/SKR/Boeun/2017 (O BE) [15], which is a Korean isolate, was used in this study. FMDV O BE was inoculated in BHK21 suspension cells at a multiplicity of infection of 0.005 and incubated at 37 °C in a 5% CO_2_ shaking incubator at 110 rpm. Subsequently, CVIS was harvested via centrifugation (4000× *g*, 20 min) at 16 h post-infection and inactivated by the addition of 3 mM binary-ethylenimine (BEI) (Sigma-Aldrich, St. Louis, MO, USA). The CVIS was then incubated in a shaking incubator at 26 °C for 24 h. Residual BEI was quenched using 2% sodium thiosulfate (Daejung Chemicals, Siheung-si, Korea). The inactivated CVIS was used by itself or concentrated ten times (10×) using a tangential flow filtration system (Merck KGaA, Darmstadt, Germany) with a 100 kDa molecular weight cut-off filter to analyze the proteins and dsDNA contamination of the target peak fractions. Otherwise, inactivated CVIS was concentrated up to 50 times (50×) by mixing it with a final concentration of 7.5% (*w/v*) PEG 6000 (Sigma-Aldrich) and 0.5 M NaCl (Sigma-Aldrich). PEG-P was obtained by centrifugation (10,000× *g* for 30 min). It was diluted to the original concentration (1×) or 10 times (10×) of the original concentration by Tris-KCl buffer variant with a low salt concentration (20 mM Tris, 150 mM KCl) to allow benzonase to work effectively.

### 2.2. Pretreatments

Both CVIS and PEG-P samples were used for the three kinds of pretreatment: chloroform extraction (C+), benzonase digestion (B+), and combinational pretreatment with chloroform and benzonase (C+B+). A non-pretreated control (NPC) sample was also prepared. For the C+ sample, the samples were vigorously mixed with the same volume of chloroform (Sigma-Aldrich). The mixture was then centrifuged at 4000× *g* for 15 min at 4 °C. The upper aqueous layer was carefully collected. For the B+ sample, samples were treated with benzonase (Sigma-Aldrich) at a final concentration of 0.025 units/μL, followed by shaking incubation at 37 °C for 1 h. After digestion, the samples were centrifuged at 16,000× *g* for 10 min at 4 °C to obtain a clear supernatant. For the C+B+ sample, chloroform extraction was conducted as C+ sample preparation, and its aqueous supernatant was used for benzonase digestion as B+ sample preparation.

### 2.3. 146S Particle Quantification with Fractionation

The quantification with the fractionation of 146S particles was performed using either SDG ultracentrifugation or SE-HPLC. For SDG ultracentrifugation, 2 mL of the sample solution was layered onto 11 mL of 15–45% sucrose density gradient tubes and ultra-centrifuged at 100,000× *g* for 4 h at 4 °C using an SW41Ti rotor (Beckman Coulter, Brea, CA, USA). The ultra-centrifuged SDG was fractionated using a continuous density gradient fractionator (Teledyne ISCO, Lincoln, NE, USA), and the absorbance of each fraction at 254 nm was recorded using a spectrophotometer component of the instrument. The area under the peak for specific fractions was measured to calculate the quantity of 146S antigens (µg/mL) according to a previous study [16]. In the case of SE-HPLC, the analysis was performed on a TSKgel G4000PWXL (300 mm × 7.8 mm I.D.) column (TOSOH Bioscience, Tokyo, Japan) combined with a TSKgel PWXL Guardcol (40 mm × 6.0 mm) guard column (TOSOH Bioscience, Tokyo, Japan) using an Agilent 1260 Infinity II system (Agilent Technologies, Santa Clara, CA, USA), composed of a quaternary pump with an online degasser, autosampler with a sample cooler, a fraction collector, a thermostatic column compartment, and a variable wavelength detector operating at 254 nm [11]. The mobile phase was composed of 30 mM Tris-HCl and 400 mM NaCl (pH 8.0), and the flow rate was set at 0.5 mL/min. The area under the target peak was integrated using the OpenLAB CDS ChemStation software, and the quantity of 146S antigens (µg/mL) was calculated according to a previous study [11]. Target peak fractions were collected by time-based fractionation from 13 to 15 min.

### 2.4. SDS-PAGE and Western Blot Analysis

Target peak fractions of pretreated CVIS (10×) and PEG-P (10×) were collected from either SDG ultracentrifugation or SE-HPLC and were used for SDS-PAGE with silver staining or Western blot analysis against FMDV VP1. Samples were mixed with 4× lithium dodecyl sulfate sample buffer (Invitrogen, Carlsbad, CA, USA) containing a sample reducing agent (Invitrogen) and boiled at 95 °C for 10 min. Proteins were separated into 4–12% Bis–Tris gels (Invitrogen). Silver staining was conducted using the EzWay Protein-Silver staining kit (KOMA Biotech, Seoul, South Korea) following the manufacturer’s instructions. The gel was transferred onto a polyvinylidene fluoride membrane (Invitrogen) using the iBlot Gel Transfer Device (Invitrogen). The membranes were blocked with 2% skim milk in phosphate-buffered saline (PBS) containing 0.1% Tween 20 (PBS-T) for an hour at room temperature with shaking, washed three times with PBS-T for 10 min, and then incubated with a house-made primary antibody for FMDV VP1 at 4 °C overnight. The next day, the membranes were washed three times with PBS-T and incubated with HRP-conjugated goat anti-mouse secondary antibody (Invitrogen) for an hour at room temperature. The antibody–antigen complexes were visualized with electrochemiluminescence Western blotting substrate (Amersham, Buckinghamshire, UK) using the Azure C600 device (Azure Biosystems, Dublin, CA, USA).

### 2.5. dsDNA Quantification

Target peak fractions of pretreated CVIS (10×) and PEG-P (10×) were collected from either SDG ultracentrifugation or SE-HPLC and were used for dsDNA quantification. Non-pretreated CVIS (10×) and PEG-P (10×) samples were also subjected to dsDNA quantification. Quantification of dsDNA was conducted using the Quant-iT PicoGreen dsDNA assay kit (Invitrogen). The dsDNA removal rate (%) was calculated as follows: dsDNA concentration of target peak fraction of respective pretreatment group/dsDNA concentration of non-pretreated sample × 100. 

### 2.6. Pure 146S Antigen Preparation and Spiking Test

Pure 146S antigens were prepared by sequential purification with SDG ultracentrifugation followed by the SE-HPLC fractionation of the concentrated SDG peak fraction. Although antigens were proven to be pure by transmission electron microscopy (data not shown), there was a slight difference between the absolute value of the pure antigen quantitated by SDG ultracentrifugation and which was quantitated by SE-HPLC. Therefore, the concentrations of pure 146S antigens used in spiking tests were calculated using the same method of quantitation. For CVIS, non-pretreated CVIS itself (1×) or C+B+ pretreated CVIS (1×) was quantitated by either SDG ultracentrifugation or SE-HPLC with or without the addition of pure 146S antigen. For PEG-P, PEG-P samples diluted to the original concentration by Tris-KCl buffer variant with low salt concentration (20 mM Tris, 150 mM KCl) itself (1×) or B+ pretreated PEG-P (1×) were quantitated by either SDG ultracentrifugation or SE-HPLC with or without the addition of pure 146S antigen. For both CVIS and PEG-P, mock samples were also prepared by heating them at 60 °C for 2 h before pure antigen spiking to verify background signals and the quantity of the spiked antigens.

### 2.7. Statistical Analysis 

Unless otherwise stated, all values are presented as the mean ± standard deviation. All experiments were performed in triplicate. Statistical analyses were performed using one-way analysis of variance followed by Tukey’s honest significant difference post hoc tests for multiple comparisons using GraphPad Prism 5 (GraphPad Software, San Diego, CA, USA). Groups that did not share a letter were significantly different (*p* < 0.05).

## 3. Results

### 3.1. Necessity of Pretreatments for the Removal of Interfering Substances in the Unpurified Upstream Sample

**Figure 1:**
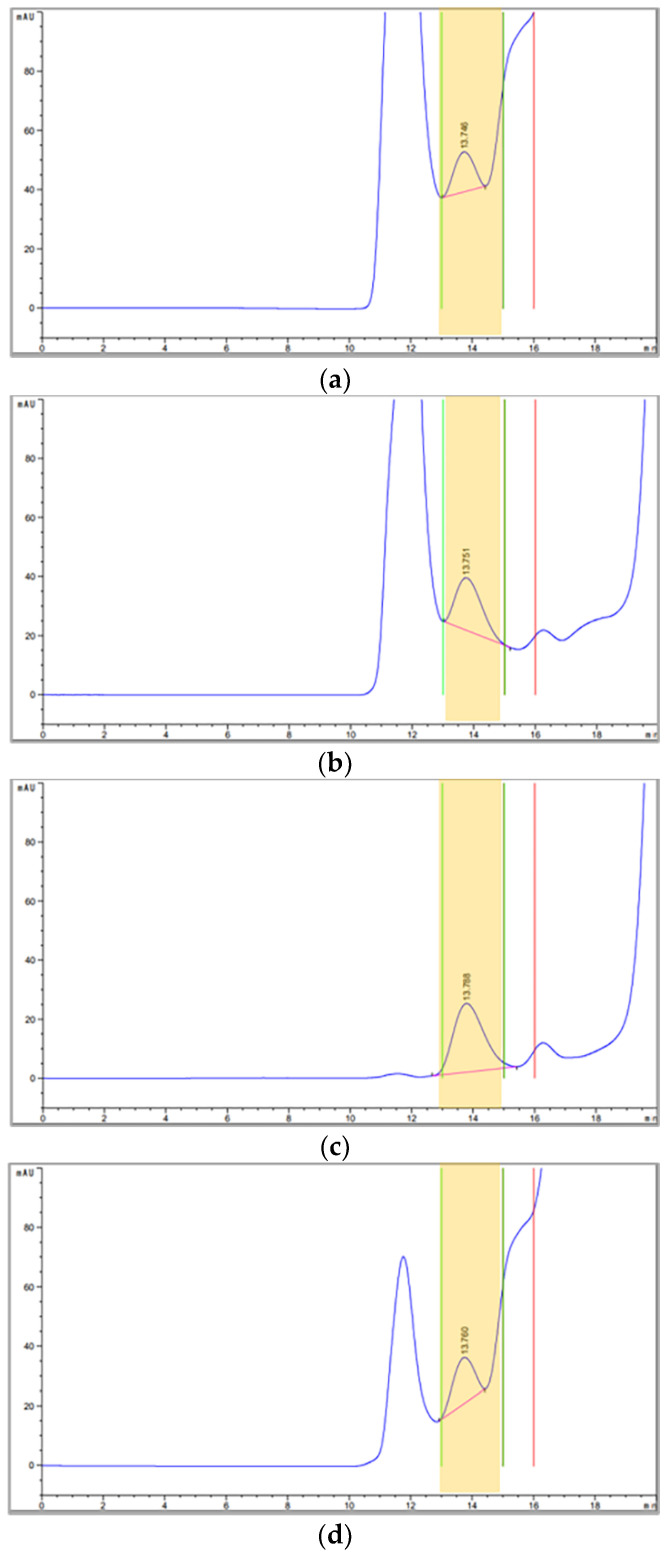

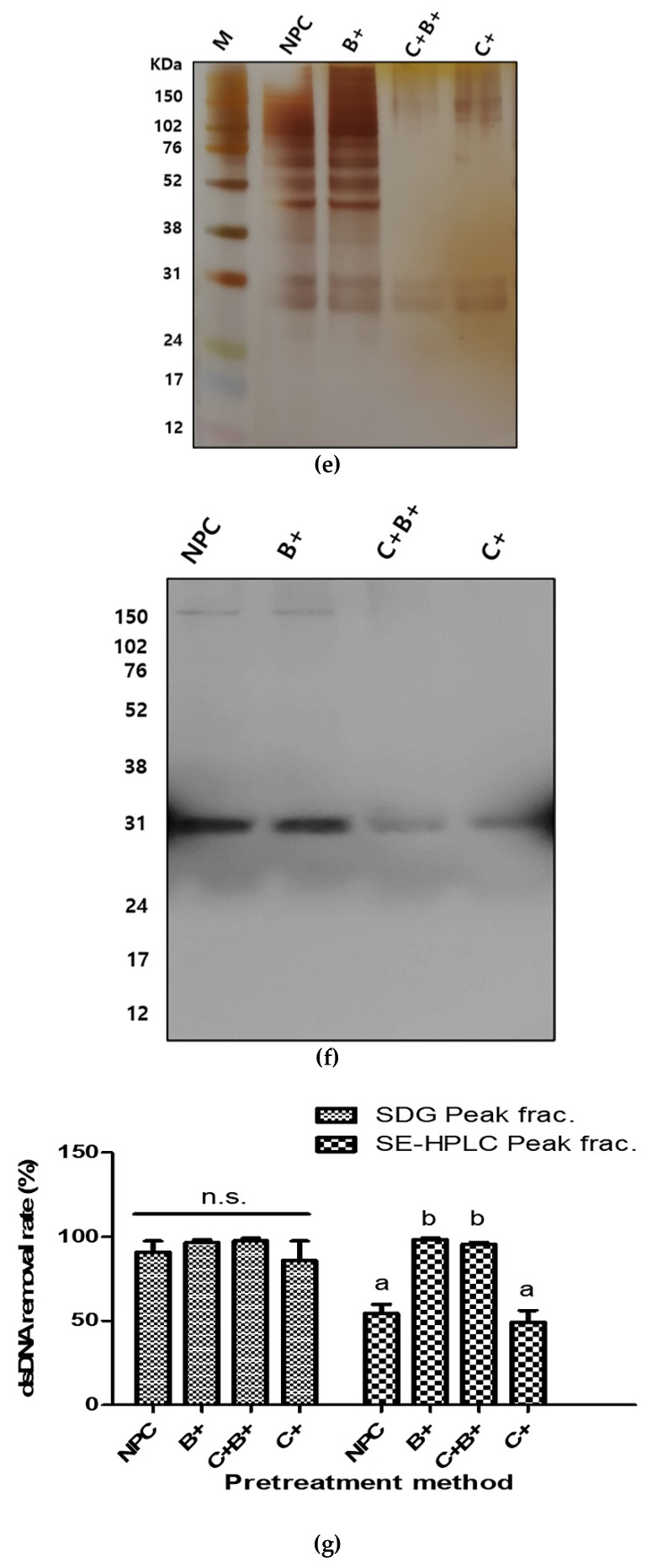
Purity of 146S antigen peak fractions collected by SE-HPLC from the 10× concentrates for the crude virus infection supernatant (CVIS) of FMDV O SKR/Boeun/2017: (**a**) original chromatograms from the SE-HPLC of CVIS (10×) without pretreatment (NPC); (**b**) original chromatogram from SE-HPLC of CVIS (10×) with benzonase digestion (B+); (**c**) original chromatogram from SE-HPLC of CVIS (10×) with combinational pretreatment of chloroform and benzonase (C+B+); (**d**) original chromatogram from SE-HPLC of CVIS (10×) with chloroform extraction (C+). Yellow backgrounds indicate the collected target peak fractions; (**e**) a silver-stained gel after SDS-PAGE of SE-HPLC target peak fraction dependent on each pretreatment method; (**f**) Western blot result against FMDV type O VP1 on SE-HPLC target peak fraction dependent on each pretreatment method; (**g**) dsDNA removal rate (%) of each target peak fraction of CVIS (10×) pretreated with various methods. Groups that do not share a letter are significantly different (*p* < 0.05). Abbreviations: SE-HPLC, size-exclusion high-performance liquid chromatography; M, marker.

CVIS (10×) samples of FMDV O BE were quantitated and fractionated by either SE-HPLC (Figure 1a–d) or SDG ultracentrifugation (Figure S1a) following respective pretreatment. The neighboring noise peaks of CVIS in the HPLC chromatogram disappeared after combined pretreatments with chloroform and benzonase. Benzonase digestion eliminated the posterior noises of the target peak, and chloroform extraction reduced the anterior noise of the target peak. Both pretreatments synergistically mitigated interfering signals. Even target peak fractions containing 146S particles of FMDV could also have several non-target materials. When the target peak fractions were analyzed by SDS-PAGE, NPC or B+ samples showed a lot of non-target protein contamination in the target peak fraction regardless of the quantitation method (Figure 1e and Figure S1b). With the addition of chloroform, both C+B+ and C+ samples exhibited the clearance of non-target protein bands except for the FMDV structural proteins, regardless of the quantitation method, even though the structural protein bands did not show the strongest signal intensity in silver staining (Figure 1e and Figure S1b). Among the structural proteins, VP1 was identified as a representative by Western blot at approximately 31 KDa (Figure 1f and Figure S1c). Meanwhile, the removal rate of host cell-derived dsDNA contamination in the target peak fraction was significantly lower in the NPC and C+ samples of SE-HPLC fractions, although the B+ and C+B+ samples exhibited a dsDNA removal rate of more than 95%, regardless of the quantitation method (Figure 1g).

### 3.2. Less Requirement of Pretreatments for the Removal of Interfering Substances in the Semi-Purified Downstream Sample

**Figure 2:**
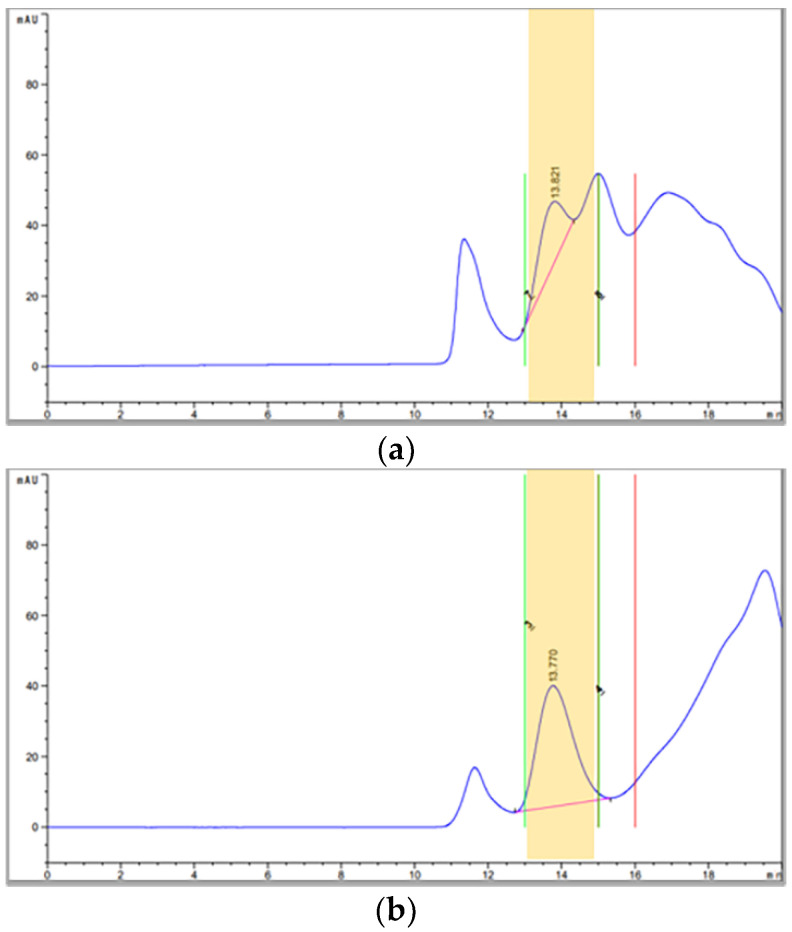

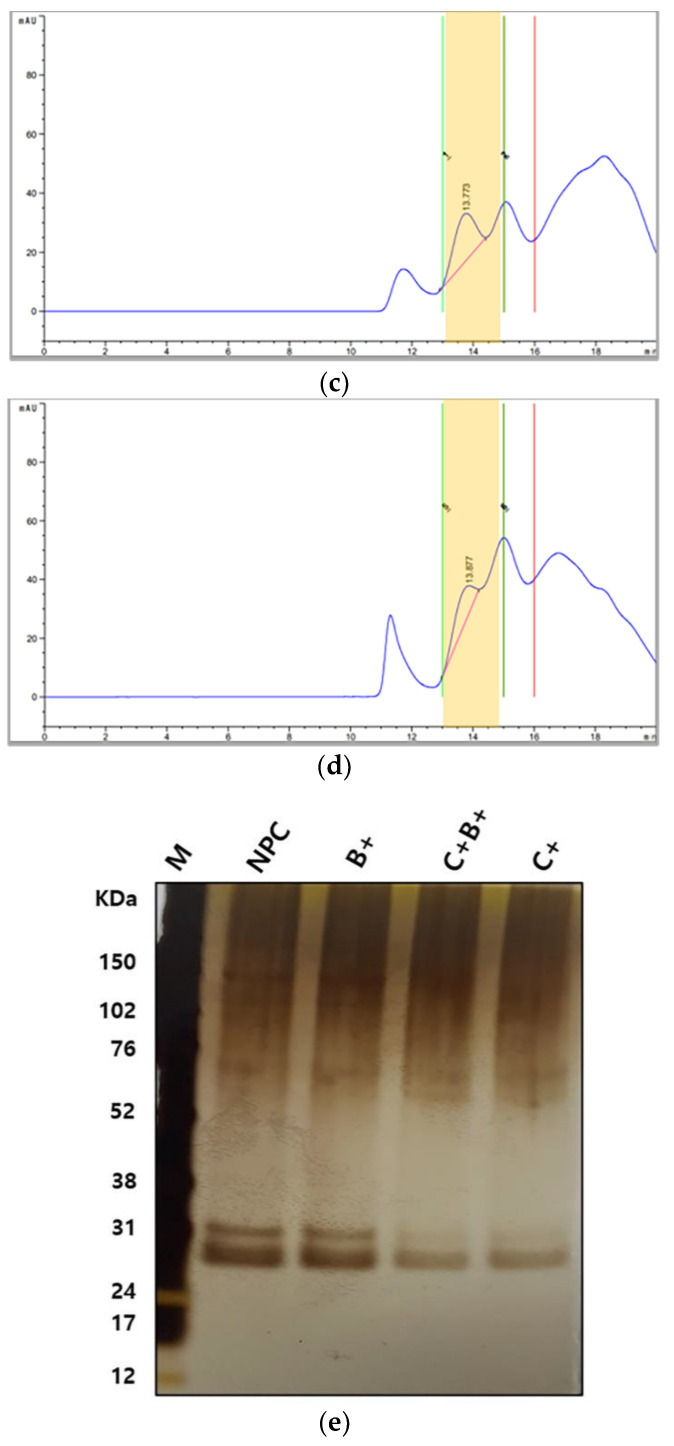

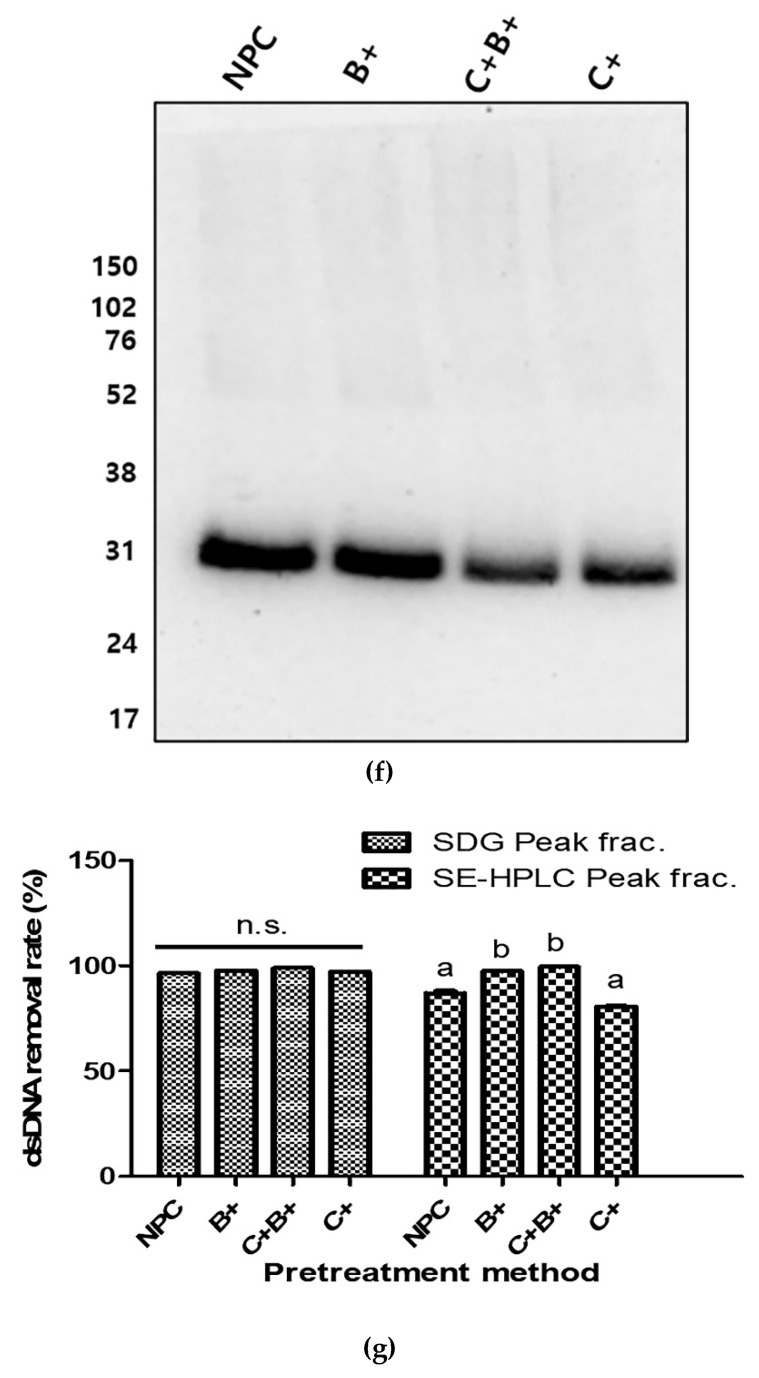
Purity of 146S antigen peak fractions collected by SE-HPLC from the 10× concentrates for PEG precipitate (PEG-P) of FMDV O SKR/Boeun/2017: (**a**) original chromatograms from SE-HPLC of PEG-P (10×) without pretreatment (NPC); (**b**) Original chromatogram from SE-HPLC of PEG-P (10×) with benzonase digestion (B+); (**c**) original chromatogram from SE-HPLC of PEG-P (10×) with combinational pretreatment of chloroform and benzonase (C+B+); (**d**) original chromatogram from SE-HPLC of PEG-P (10×) with chloroform extraction (C+). Yellow backgrounds indicate the collected target peak fractions; (**e**) a silver-stained gel after SDS-PAGE of SE-HPLC target peak fraction dependent on each pretreatment method; (**f**) Western blot result against FMDV type O VP1 on SE-HPLC target peak fraction dependent on each pretreatment method; (**g**) dsDNA removal rate (%) of each target peak fraction of PEG-P (10×) pretreated with various methods. Groups that do not share a letter are significantly different (*p* < 0.05). Abbreviations: SE-HPLC, size-exclusion high-performance liquid chromatography; M, marker.

PEG-P (10×) samples of FMDV O BE were quantitated and fractionated by either SE-HPLC (Figure 2a–d) or SDG ultracentrifugation (Figure S2a) following respective pretreatment. The neighboring noise peaks of PEG-P in the HPLC chromatogram disappeared dramatically after benzonase digestion. Chloroform or combinational pretreatment (chloroform + benzonase) did not eliminate interfering peaks. Even target peak fractions containing 146S particles of FMDV could also have several non-target materials. In contrast to the HPLC analysis of PEG-P, background noise peaks were not observed even in the non-pretreated sample by SDG ultracentrifugation (Figure S2a); when the target peak fractions, containing 146S particles of FMDV, were analyzed by SDS-PAGE, all samples, even NPC, showed distinct protein bands at approximately 31 KDa without the significant contamination of non-target protein bands in the target peak fraction, regardless of the quantitation method (Figure 2e and Figure S2b). Although both C+B+ and C+ samples from SDG ultracentrifugation appeared to be slightly purer than the NPC or B+ samples (Figure S2b), those from SE-HPLC did not show noticeable differences in their purity from NPC or B+ samples (Figure 2e). Among the structural proteins, VP1 was identified as a representative by Western blot at approximately 31 KDa (Figure 2f and Figure S2c). Meanwhile, the removal rate of host cell-derived dsDNA contamination in the target peak fraction was over 95% from SDG ultracentrifugation, while both NPC and C+ samples collected by SE-HPLC displayed a slightly lower removal rate of less than 90% (Figure 2g).

### 3.3. Validity of the Pretreatment Method for CVIS

As CVIS contains abundant non-target proteins and host cell-derived dsDNA even in their target peak fractions, combined pretreatment with chloroform and benzonase was required. To validate whether the pretreatment method was effective, pure 146S antigen spiking tests were conducted. In the case of the non-pretreated sample, CVIS itself was already difficult to quantitate because of the high degree of background signal (Figures S3 and S4). Therefore, there were more than 100% gaps between the non-pretreated and C+B+ CVIS-only samples (Table 1 and Table 2). Furthermore, although the antigen concentration of the heated CVIS + pure 146S antigen sample should theoretically be the same as that of spiked antigen, non-pretreated samples showed highly overestimated values by SDG quantitation (Table 1) or highly underestimated values by SE-HPLC quantitation (Table 2). Meanwhile, non-pretreated CVIS with pure 146S antigen spiking samples showed quite different quantitation values between theoretical estimation, drawn by the sum of known concentrations of spiked antigen and measured concentrations of the CVIS only sample, and practical estimation, drawn by the sum of the practically measured concentrations of the spiked antigen mixed with heated CVIS and that of the CVIS only sample, regardless of the quantitation method (Table 1 and Table 2). However, this inaccuracy could be solved by C+B+ pretreatment as the error rates of the CVIS + pure 146S antigen sample were less than 5% by SDG quantitation (Table 1) and less than 3% by SE-HPLC quantitation (Table 2), respectively. 

### 3.4. Validity of the Pretreatment Method for PEG-P 

As PEG-P samples were already proven to be semi-purified as their target peak fraction contained a negligible degree of non-target proteins and a small amount of host cell-derived dsDNA even in NPC (Figure 2e,g and Figure S2b), and the chloroform pretreatment seemed to induce a slight loss of 146S antigens (Figure 2f and Figure S2c), the single use of benzonase was tested to determine whether the pretreatment could enhance the quantitation accuracy of a pure 146S antigen spiking test. There was little background signal interfering with the target peak in both SDG quantitation and SE-HPLC quantitation (Figures S5 and S6). Consequently, there was no significant difference between the quantitated value of the non-pretreated sample and that of the B+ sample in every group, regardless of the quantitation method (Table 3 and Table 4). SDG quantitation showed highly similar values regardless of the pretreatment (Table 3), while SE-HPLC quantitation exhibited slightly enhanced accuracy by benzonase digestion because non-pretreated PEG-P with pure 146S antigen spiking samples exhibited error rates of approximately 9%; however, the benzonase-pretreated PEG-P with pure 146S antigen-spiking samples displayed error rates of less than 5% (Table 4). 

## 4. Discussion

Foot-and-mouth disease (FMD) is a viral disease with high contagiousness that threatens a lot of the livestock industry. The disease is controlled by existing vaccines. The efficacy of the vaccine is determined by its content in 146S particles that is represented by the intact virion of the virus. The 146S particles are very unstable and easily dissociate into less immunogenic particles, which is why the identification and quantification of 146S particles are critical in the process of vaccine production. The standard method for 146S particles’ quantification is sucrose density gradient (SDG). This method is very complex, needs a lot of preparation steps and is time-consuming, which is why new methods need to be validated for FMD vaccine quality control. Size-exclusion high-performance liquid chromatography (SE-HPLC) is a recommended alternative method with high specificity, repeatability and accuracy, but need proper sample pretreatment according to the production process phase.

Generalized FMD vaccine antigen quantitation methods, such as SDG fractionation and SE-HPLC, are all based on the UV absorbance of viral genomic RNA at 254 nm, considering that the extinction coefficient of FMDV 146S particles is 72 [12]. Therefore, every substance that has UV absorbance at 254 nm can create interfering signals that hamper the exact quantitation of 146S antigens. Because gel columns utilized in SE-HPLC have resins consisting of a porous matrix of spherical particles that lack any specific binding properties [17], the proper pretreatment of the analytical sample is required in size exclusion chromatography more than other types of chromatography, such as ion-exchange chromatography or affinity chromatography, to separate the target material from non-interested contaminants.

FMDV, which belongs to the Aphthovirus genus of Picornaviridae, encodes viroporins in its genome [18]. During the late phase of virus infection, the accumulation of viroporins induces a progressive increase in cellular membrane permeability followed by host cell lysis [19]. As a consequence of cell lysis, mature virions and various intracellular components of host cells are released. Thus, CVIS samples contain abundant interfering substances, as shown in Figure 1 and Figure S4. As the analytical wavelength of the present study was set at 254 nm, free nucleic acids, both RNAs and DNAs, would most potently interfere with the target signal. For this reason, previous studies have usually focused on the enzymatic digestion of nucleic acids only [10,11,14]. However, non-specific host proteins, particularly those with high molecular weights, could partly interfere with the target signal in the SE-HPLC analysis of FMD vaccine antigens, although their maximum absorbance wavelength would be at 280 nm [20]. 

A previous study compared three pretreatment methods, including ultracentrifugation, PEG precipitation, and nuclease digestion, for the quantification of the 146S antigen in foot-and-mouth disease vaccines by SE-HPLC and reported that benzonase digestion was the best of the tested methods [14]. However, abundant impurities might have already been removed from their samples during consecutive production processes, because the authors only focused on the aqueous samples extracted from the demulsification of complete vaccine products [14]. 

Another previous study reported that benzonase digestion was adequate for the CVIS samples and additional chloroform extraction was required for the PEG-P samples [11]; however, the sole pretreatment of benzonase in CVIS could not remove a variety of non-specific host proteins, although those samples were primarily purified by collecting target peak fractions from either SE-HPLC or SDG fractionation (Figure 1e and Figure S1b). In addition, chromatograms of the CVIS (B+) sample showed the low resolution of the target peak in both SE-HPLC and SDG fractionation (Figure 1b and Figure S1a), while that of CVIS (C+B+) showed the highest resolution of the target peak (Figure 1c and Figure S1a). As shown in the SE-HPLC chromatogram in Figure 1, benzonase digestion removed the noise at the posterior part of the target peak (approximately 16 min of retention time), while the chloroform extraction removed the noise at the anterior part of the target peak (approximately 11 min of retention time). Therefore, the combined use of the two pretreatment methods distinctly improves the resolution of the target peak by removing the adjacent interfering signals synergistically. Despite the fact that there seemed to be an inevitable loss of 146S antigens through the sequential pretreatment of chloroform and benzonase (Figure 1f and Figure S1c), this amount was negligible in CVIS, as verified in the pure antigen spiking test (Table 1 and Table 2).

Meanwhile, the combined pretreatment of chloroform and benzonase was not the optimal pretreatment method for more purified downstream samples such as PEG-P. Because PEG-P samples already had few non-specific host proteins in their target peak fractions collected by either SE-HPLC or SDG fractionation (Figure 2e and Figure S2b), further chloroform extraction destabilized the 146S antigens and increased the loss (Figure 2f and Figure S2a,c). The resolution of the target peak was also highest in the PEG-P (B+) chromatogram (Figure 2b). In practice, it was proven that there was less necessity for pretreatment in PEG-P samples if concentrated samples were diluted to their original concentration (Table 3 and Table 4). However, the benzonase digestion step could be added to quantify concentrated PEG-P samples, if needed (Figure 2b). The current manuscript presents an interesting study intended to identify the correct pretreatment method for the SE-HPLC analysis of 146S particles from the FMD vaccine for each phase of production. This work is very useful for the research community as a rapid specific method needs to be validated for each step of vaccine production. There is a lack of scientific reference regarding the pretreatment methods for the SE-HPLC analysis of 146S particles from the FMD vaccine and this work can provide important scientific information on the topic. To the best of our knowledge, this was the first report that combined pretreatment with chloroform and benzonase could dramatically reduce interfering materials for the quantification of the 146S antigen in the CVIS by HPLC analysis. Although further studies for the differential quantification of 146S antigens from empty particles would be required, SE-HPLC analysis enables convenient, rapid and trustworthy in-process quality control during the FMD vaccine production process—if the samples could be prepared by optimized pretreatment methods.

In conclusion, the current study not only validated the necessity of proper pretreatment for the accurate quantification of FMD vaccine antigens using an automated instrument (SE-HPLC), but could also be utilized for the preparation of quantification samples for classic SDG fractionation or the development of new quantification technologies, giving them higher reliability. 

## Figures and Tables

**Table 1:** A spiking test result by SDG ultracentrifugation for the validation of the pretreatment method for CVIS.

Pretreatment	Sample Information		SDG Quantitation (μg/mL)	Theoretical Estimation (μg/mL)	Theoretical % Recovery	Practical Estimation (μg/mL)	Practical % Recovery
	Condition	Spiked Ag (μg/mL)					
None	CVIS only	0	1.77 ± 0.19 ^b^	-	-	-	-
	CVIS + pure 146S Ag	4.89 ^a^	13.59 ± 0.39 ^c^	6.66 ± 0.19 ^f^	204.3 ± 11.6 ^g^	9.52 ± 0.69 ^h^	143.5 ± 14.7 ^i^
	Heated CVIS (60 °C, 2 h)	0	0 ^d^	-	-	-	-
	Heated CVIS + pure 146S Ag	4.89 ^a^	7.75 ± 0.52 ^e^	4.89 ^f^	158.5 ± 10.6 ^g^	-	-
C+B+	CVIS only	0	3.70 ± 0.27 ^b^	-	-	-	-
	CVIS + pure 146S Ag	4.89 ^a^	8.96 ± 0.61 ^c^	8.59 ± 0.27 ^f^	104.3 ± 6.0 ^g^	9.14 ± 0.24 ^h^	98.1 ± 7.0 ^i^
	Heated CVIS (60 °C, 2 h)	0	0 ^d^	-	-	-	-
	Heated CVIS + pure 146S Ag	4.89 ^a^	5.44 ± 0.14 ^e^	4.89 ^f^	111.2 ± 2.8 ^g^	-	-

^a^ Known amount of spiked pure antigens; ^b^ practically quantitated 146S antigen concentration in the CVIS (1×) sample before spiking; ^c^ practically quantitated 146S antigen concentration of CVIS (1×) sample after spiking; ^d^ practically quantitated 146S antigen concentration in heated CVIS (1×) sample before spiking; ^e^ practically quantitated 146S antigen concentration in heated CVIS (1×) samples after spiking; ^f^ ‘a + b’ for unheated sample or ‘a + d’ for heated sample; ^g^ ‘c/f × 100’ for unheated sample or ‘e/f × 100’ for heated sample; ^h^ b + e; ^i^ c/h × 100. Abbreviations: CVIS, crude virus infection supernatant; SDG, sucrose density gradient; C+B+, combined pretreatment with chloroform and benzonase; Ag, antigen.

**Table 2:** A spiking test result by SE-HPLC for the validation of the pretreatment method for CVIS.

Pretreatment	Sample Information		SE-HPLC Quantitation (μg/mL)	Theoretical Estimation (μg/mL)	Theoretical % Recovery	Practical Estimation (μg/mL)	Practical % Recovery
	Condition	Spiked Ag (μg/mL)					
None	CVIS only	0	1.24 ± 0.02 ^b^	-	-	-	-
	CVIS + pure 146S Ag	5.99 ^a^	5.59 ± 0.01 ^c^	7.23 ± 0.02 ^f^	77.3 ± 0.2 ^g^	3.95 ± 0.08 ^h^	141.6 ± 2.7 ^i^
	Heated CVIS (60 °C, 2 h)	0	0 ^d^	-	-	-	-
	Heated CVIS + pure 146S Ag	5.99 ^a^	2.71 ± 0.07 ^e^	5.99 ^f^	45.2 ± 1.1 ^g^	-	-
C+B+	CVIS only	0	3.97 ± 0.02 ^b^	-	-	-	-
	CVIS + pure 146S Ag	5.99 ^a^	10.26 ± 0.13 ^c^	9.96 ± 0.02 ^f^	102.9 ± 1.1 ^g^	9.99 ± 0.17 ^h^	102.7 ± 1.9 ^i^
	Heated CVIS (60 °C, 2 h)	0	0 ^d^	-	-	-	-
	Heated CVIS + pure 146S Ag	5.99 ^a^	6.01 ± 0.16 ^e^	5.99 ^f^	100.4 ± 2.6 ^g^	-	-

^a^ Known amount of spiked pure antigens; ^b^ practically quantitated 146S antigen concentration in the CVIS (1×) sample before spiking; ^c^ practically quantitated 146S antigen concentration of CVIS (1×) sample after spiking; ^d^ practically quantitated 146S antigen concentration in heated CVIS (1×) sample before spiking; ^e^ practically quantitated 146S antigen concentration in heated CVIS (1×) samples after spiking; ^f^ ‘a + b’ for unheated sample or ‘a + d’ for heated sample; ^g^ ‘c/f × 100’ for unheated sample or ‘e/f × 100’ for heated sample; ^h^ b + e; ^i^ c/h × 100. Abbreviations: CVIS, crude virus infection supernatant; SE-HPLC, size-exclusion high-performance liquid chromatography; C+B+, combined pretreatment with chloroform and benzonase; Ag, antigen.

**Table 3:** A spiking test result by SDG ultracentrifugation for the validation of the pretreatment method for PEG-P.

Pretreatment	Sample Information		SDG Quantitation (μg/mL)	Theoretical Estimation (μg/mL)	Theoretical % Recovery	Practical Estimation (μg/mL)	Practical % Recovery
	Condition	Spiked Ag (μg/mL)					
None	PEG-P only	0	2.86 ± 0.24 ^b^	-	-	-	-
	PEG-P + Pure 146S Ag	4.89 ^a^	7.68 ± 0.56 ^c^	7.75 ± 0.24 ^f^	99.0 ± 4.5 ^g^	8.33 ± 0.38 ^h^	92.1 ± 3.7 ^i^
	Heated PEG-P (60 °C, 2 h)	0	0 ^d^	-	-	-	-
	Heated PEG-P + pure 146S Ag	4.89 ^a^	5.47 ± 0.29 ^e^	4.89 ^f^	111.9 ± 5.9 ^g^	-	-
B+	PEG-P only	0	2.90 ± 0.01 ^b^	-	-	-	-
	PEG-P + pure 146S Ag	4.89 ^a^	7.78 ± 0.36 ^c^	7.79 ± 0.01 ^f^	100.0 ± 4.6 ^g^	8.37 ± 0.28 ^h^	93.1 ± 7.0 ^i^
	Heated PEG-P (60 °C, 2 h)	0	0^d^	-	-	-	-
	Heated PEG-P + pure 146S Ag	4.89 ^a^	5.47 ± 0.28 ^e^	4.89 ^f^	111.9 ± 5.8 ^g^	-	-

^a^ Known amount of spiked pure antigens; ^b^ practically quantitated 146S antigen concentration in PEG-P (1×) sample before spiking; ^c^ practically quantitated 146S antigen concentration in the PEG-P (1×) sample after spiking; ^d^ practically quantitated 146S antigen concentration in heated PEG-P (1×) sample before spiking; ^e^ practically quantitated 146S antigen concentration in heated PEG-P (1×) samples after spiking; ^f^ ‘a + b’ for unheated sample or ‘a + d’ for heated sample; ^g^ ‘c/f × 100’ for unheated sample or ‘e/f × 100’ for heated sample; ^h^ b + e; ^i^ c/h × 100. Abbreviations: PEG-P, PEG precipitate; SDG, sucrose density gradient; B+, benzonase digestion; Ag, antigen.

**Table 4:** A spiking test result by SE-HPLC for the validation of the pretreatment method for PEG-P.

Pretreatment	Sample Information		SE-HPLC Quantitation (μg/mL)	Theoretical Estimation (μg/mL)	Theoretical % Recovery	Practical Estimation (μg/mL)	Practical % Recovery
	Condition	Spiked Ag (μg/mL)					
None	PEG-P only	0	3.14 ± 0.84 ^b^	-	-	-	-
	PEG-P + pure 146S Ag	5.99 ^a^	9.88 ± 0.04 ^c^	9.13 ± 0.84 ^f^	108.9 ± 10.1 ^g^	9.08 ± 0.65 ^h^	109.2 ± 7.7 ^i^
	Heated PEG-P (60 °C, 2 h)	0	0 ^d^	-	-	-	-
	Heated PEG-P + pure 146S Ag	5.99 ^a^	5.94 ± 0.20 ^e^	5.99 ^f^	99.2 ± 3.4 ^g^	-	-
B+	PEG-P only	0	3.12 ± 0.06 ^b^	-	-	-	-
	PEG-P + pure 146S Ag	5.99 ^a^	9.26 ± 0.18 ^c^	9.11 ± 0.06 ^f^	101.7 ± 2.5 ^g^	8.98 ± 0.44 ^h^	103.4 ± 6.9 ^i^
	Heated PEG-P (60 °C, 2 h)	0	0 ^d^	-	-	-	-
	Heated PEG-P + pure 146S Ag	5.99 ^a^	5.86 ± 0.39 ^e^	5.99 ^f^	97.8 ± 6.5 ^g^	-	-

^a^ Known amount of spiked pure antigens; ^b^ practically quantitated 146S antigen concentration in PEG-P (1×) sample before spiking; ^c^ practically quantitated 146S antigen concentration in the PEG-P (1×) sample after spiking; ^d^ practically quantitated 146S antigen concentration in heated PEG-P (1×) sample before spiking; ^e^ practically quantitated 146S antigen concentration in heated PEG-P (1×) samples after spiking; ^f^ ‘a + b’ for unheated sample or ‘a + d’ for heated sample; ^g^ ‘c/f × 100’ for unheated sample or ‘e/f × 100’ for heated sample; ^h^ b + e; ^i^ c/h × 100. Abbreviations: PEG-P, PEG precipitate; SDG, sucrose density gradient; B+, benzonase digestion; Ag, antigen.

## Data Availability

Not applicable.

## Supplementary Materials

The following are available online at https://www.mdpi.com/article/10.3390/vaccines9111361/s1, Figure S1: Purity of 146S antigen peak fractions collected by SDG fractionation from the 10× concentrates for crude virus infection supernatant (CVIS) of FMDV O SKR/Boeun/2017, Figure S2: Purity of 146S antigen peak fractions collected by SDG fractionation from the 10× concentrates for PEG precipitate (PEG-P) of FMDV O SKR/Boeun/2017, Figure S3: Representative original chromatograms drawn by SDG fractionation of CVIS (1×) with or without spiked 146S antigens, Figure S4: Representative original chromatograms drawn by SE-HPLC of CVIS (1×) with or without spiked 146S antigens, Figure S5: Representative original chromatograms drawn by SDG fractionation of PEG-P (1×) with or without spiked 146S antigens, Figure S6: Representative original chromatograms drawn by SE-HPLC of PEG-P (1×) with or without spiked 146S antigens.
