# Supplementary material for: Validation of Pretreatment Methods for the In-Process Quantification of Foot-and-Mouth Disease Vaccine Antigens
Source: Vaccines (Basel). 2021 Nov 19;9(11):1361. doi: 10.3390/vaccines9111361 (PMC8624908; doi:10.3390/vaccines9111361)
Supplement: Supplementary file 1 [file vaccines-09-01361-s001.zip › vaccines-1459777 supplement.pdf]

Supplementary materials

Title: Validation of pretreatment methods for the in-process quantification of foot-and-mouth disease vaccine antigens

Ah-Young Kim, Sun Young Park, Sang Hyun Park, Jong Sook Jin, Eun-Sol Kim, Jae Young Kim, Jong-Hyeon Park and Young-Joon Ko*

Animal and Plant Quarantine Agency, Gimcheon, Gyeonsangbuk-do 39660, Republic of Korea

* Corresponding author: Young-Joon Ko

Center for FMD Vaccine Research, Animal and Plant Quarantine Agency, Gimcheon, Gyeonsangbuk-do 39660, Republic of Korea

Tel.: 82-54-912-0908/ Fax: 82-54-912-0890

E-mail: koyoungjoon@korea.kr

Inventory of Supplemental Information

Supplemental Figures and Legends:

- **Figure S1 (refers to Figure 1)**
- **Figure S2 (refers to Figure 2)**
- **Figure S3 (refers to Table 1)**
- **Figure S4 (refers to Table 2)**
- **Figure S5 (refers to Table 3)**
- **Figure S6 (refers to Table 4)**

Supplemental figures and legends

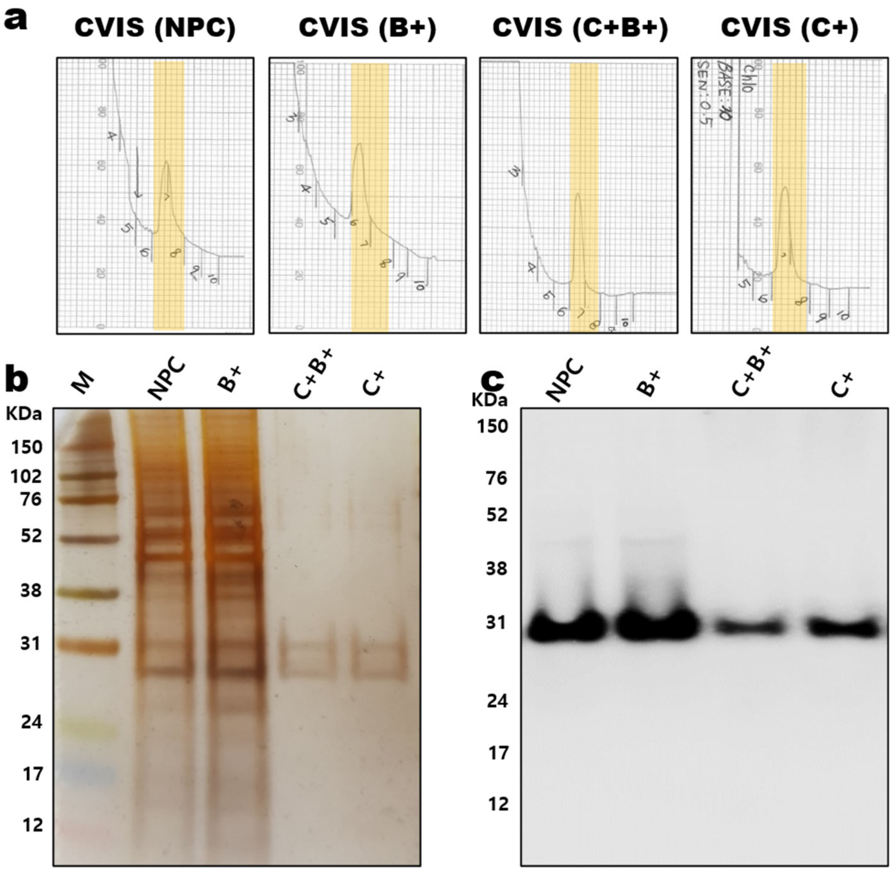

Fig. S1. Purity of 146S antigen peak fractions collected by SDG fractionation from the 10 × concentrates for crude virus infection supernatant (CVIS) of FMDV O SKR/Boeun/2017.

(a) Original chromatograms from SDG fractionation of CVIS (10×) dependent on each pretreatment method. Yellow backgrounds indicate the collected target peak fractions for further purity tests in b and c. (b) A silver-stained gel after SDS-PAGE of SDG ultracentrifugation target peak fraction dependent on each pretreatment method. (C) Western blot result against FMDV type O VP1 on SDG ultracentrifugation target peak fraction dependent on each pretreatment method.

Abbreviations: SDG, sucrose density gradient; NPC, non-pretreated control; B+, benzonase digestion; C+, chloroform extraction; C+B+, combinational pretreatment of chloroform and benzonase; M, marker.

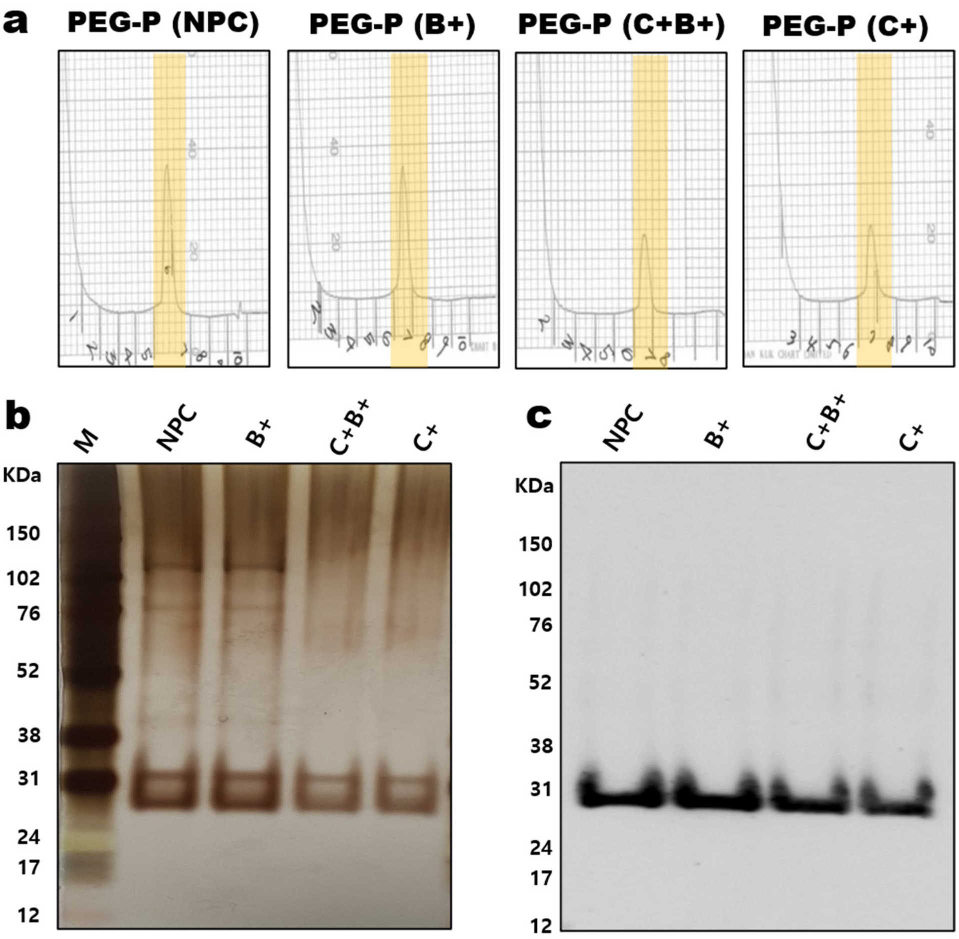

Fig. S2. Purity of 146S antigen peak fractions collected by SDG fractionation from the 10× concentrates for PEG precipitate (PEG-P) of FMDV O SKR/Boeun/2017.

(a) Original chromatograms from SDG fractionation of PEG-P (10×) dependent on each pretreatment method. Yellow backgrounds indicate the collected target peak fractions for further purity tests in B and C. (b) A silver-stained gel after SDS-PAGE of SDG ultracentrifugation target peak fraction dependent on each pretreatment method. (c) Western blot result against FMDV type O VP1 on SDG ultracentrifugation target peak fraction dependent on each pretreatment method.

Abbreviations: SDG, sucrose density gradient; NPC, non-pretreated control; B+, benzonase digestion; C+, chloroform extraction; C+B+, combinational pretreatment of chloroform and benzonase; M, marker.

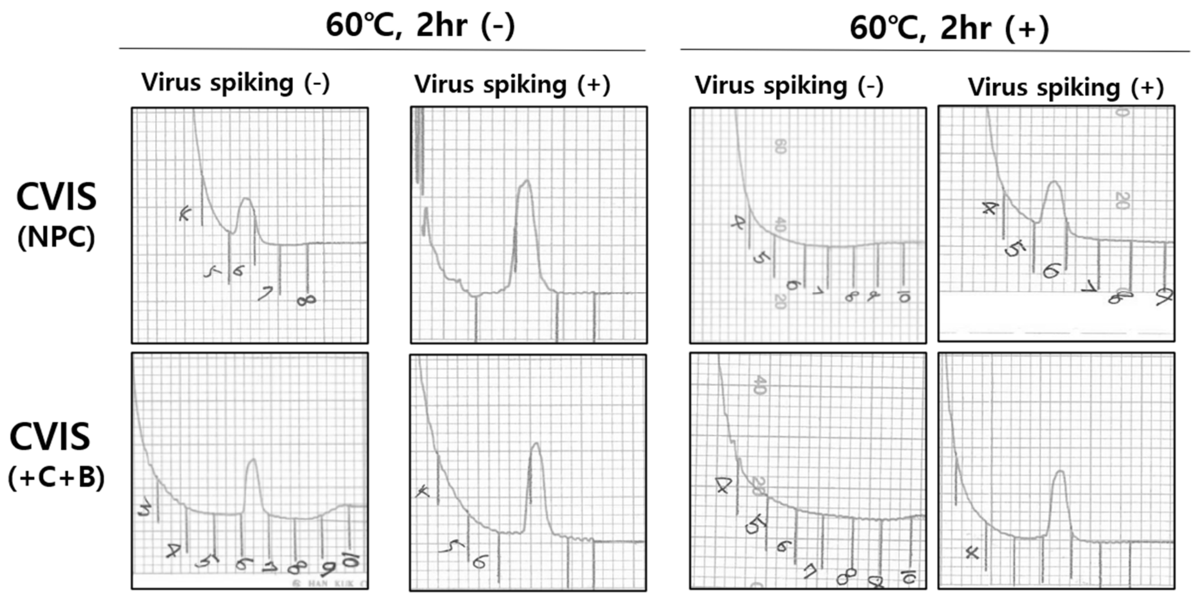

Fig. S3. Representative original chromatograms drawn by SDG fractionation of CVIS (1×) with or without spiked 146S antigens.

Abbreviations: CVIS, crude virus infection supernatant; SDG, sucrose density gradient; NPC, non-pretreated control; C+B+, combinational pretreatment of chloroform and benzonase.

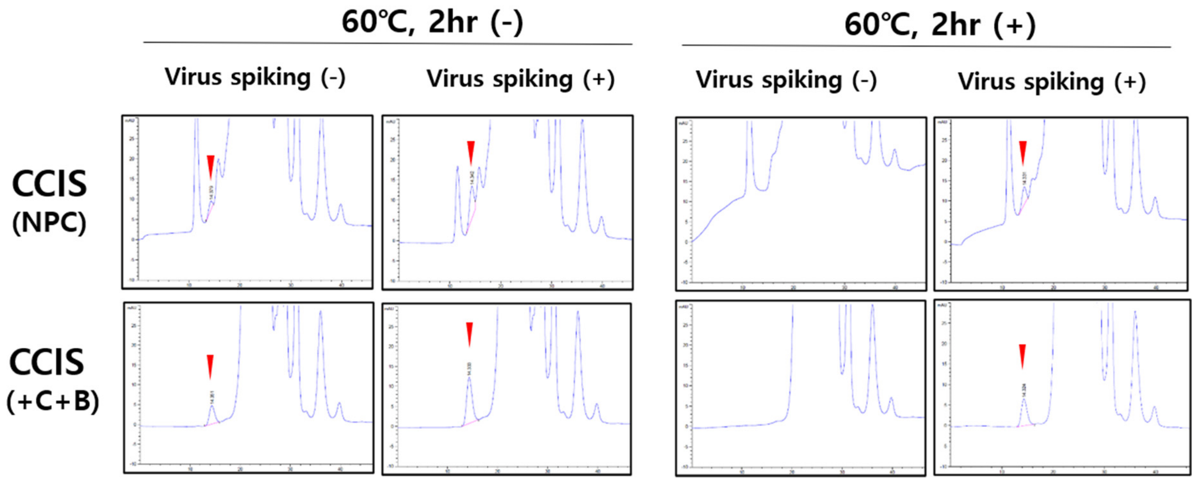

Fig. S4. Representative original chromatograms drawn by SE- HPLC of CVIS (1×) with or without spiked 146S antigens. Red arrowheads indicate the FMDV 146S antigen peaks.

Abbreviations: CVIS, crude virus infection supernatant; SE-HPLC, size-exclusion high-performance liquid chromatography; NPC, non-pretreated control; C+B+, combinational pretreatment of chloroform and benzonase.

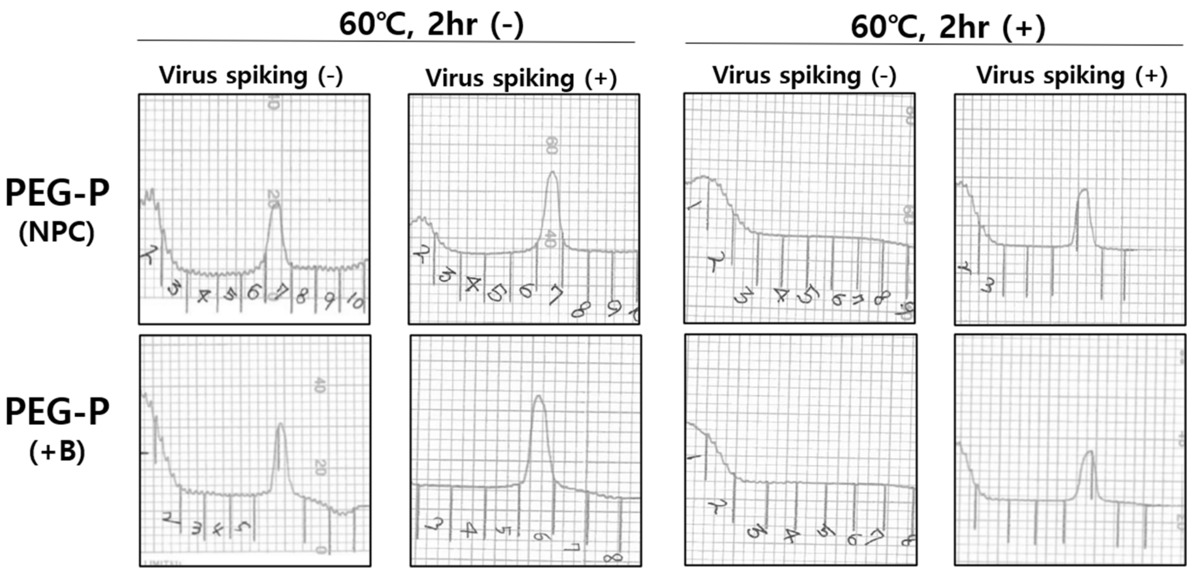

Fig. S5. Representative original chromatograms drawn by SDG fractionation of PEG-P (1×) with or without spiked 146S antigens.

Abbreviations: PEG-P, polyethylene glycol-precipitate; SDG, sucrose density gradient; NPC, non-pretreated control; B+, benzonase digestion.

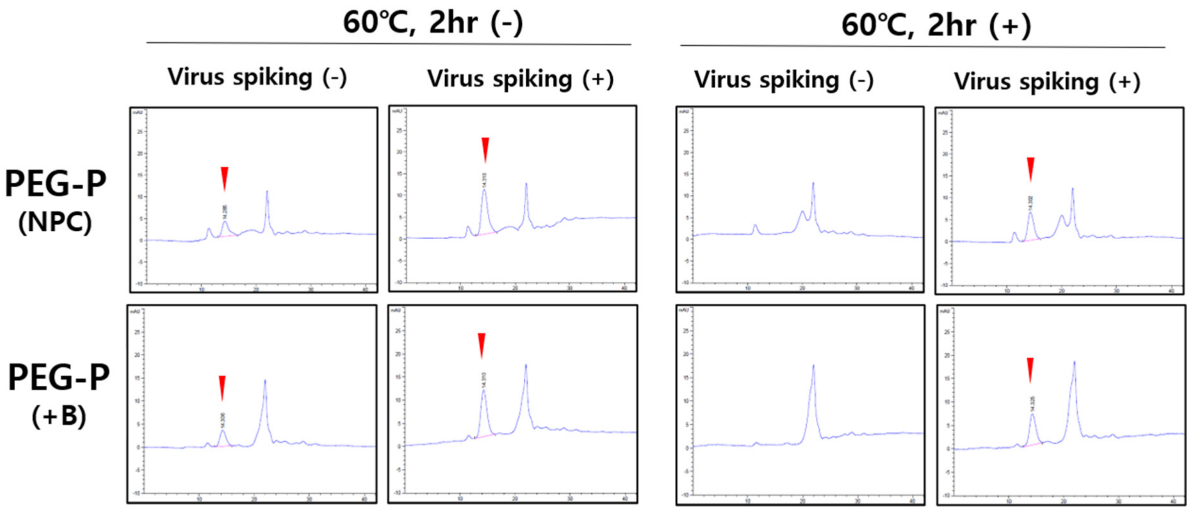

Fig. S6. Representative original chromatograms drawn by SE- HPLC of PEG-P (1×) with or without spiked 146S antigens. Red arrowheads indicate the FMDV 146S antigen peaks.

Abbreviations: PEG-P, polyethylene glycol-precipitate; SE-HPLC, size-exclusion high-performance liquid chromatography; NPC, non-pretreated control; B+, benzonase digestion.
